# Evidence of Language Development Using Brief Animated Stimuli: A Systematic Review

**DOI:** 10.3390/brainsci14020150

**Published:** 2024-01-31

**Authors:** Triantafyllia I. Vlachou, Maria Kambanaros, Panagiotis Plotas, Voula Chris Georgopoulos

**Affiliations:** 1 Department of Speech and Language Therapy, University of Patras, 26504 Patras, Greece; 2 The Brain and Neurorehabilitation Lab, Department of Rehabilitation Sciences, Cyprus University of Technology, Limassol 3041, Cyprus; 3 Primary Health Care Laboratory, School of Health Rehabilitation Sciences, University of Patras, 26504 Patras, Greece

**Keywords:** animation, language development, typically developing children, speech language pathology

## Abstract

There is limited evidence regarding the effect of animation compared to static pictures on children’s language development. The aim was to systematically review the available literature for evidence concerning the effect of brief animation on spoken language responses (receptive—listening or expressive—speaking) in typically developing (TD) children aged 3 to 9 years. Five databases were searched, resulting in seven included studies. The characteristics of animated stimuli, the manner of presentation, and the language-related tasks were recorded, and questions were posed about the effect of brief animation on children’s receptive and expressive language abilities. The evidence suggests that animation may have a positive effect on expressive language abilities of children compared to static pictures. As far as the effect of animation on receptive language performance is concerned, the evidence is less concrete. Future directions regarding the potential of animation on language development are discussed.

## 1. Introduction

Limited research has been conducted on the impact of animations compared to static pictures on children’s language development within the field of speech language pathology (SLP). This systematic review aimed to identify studies employing brief animations to explore receptive and expressive verbal language responses in typically developing children (TD) aged 3 to 9 years. The objective was to investigate any positive or negative effects of brief animation on language development and related non-verbal behaviors (e.g., motivation, distraction, and concentration).

### 1.1. What Is Animation?

Modern technology offers diverse animation forms (e.g., 2D and 3D; [1]) integrated into daily life through computers and mobile devices (e.g., applications, users’ interfaces, etc.) [2,3]. However, the scope of this study is not the technological advancements of animations, but rather their potential effect on language development in TD children.

The main theoretical frameworks concerning human perception of animation are grounded in the principles of gestalt psychology [4,5,6], visual communication [6,7,8], the cognitive theory of multimedia learning [9], the cognitive load theory [10,11,12], and the animation processing model [13]. In this study, the terminology employed aligns with that of Bétrancourt and Tversky [7], who defined animation as “… any application that generates a sequence of frames, wherein each frame represents a modification of the preceding one, and where the sequencing of frames is determined by either the designer or the user” [7] (p. 313).

The existing literature has extensively explored the efficacy of animations in educational contexts, comparing them to static images for conveying complex subjects (e.g., biology and chemistry), with a focus on adolescent and young adult learners [6,7,14,15,16,17].

The use of animation in instructional materials initially gained interest due to its dynamic nature, conveying additional information regarding temporal changes. Also, animation was deemed attractive, amusing, and motivating. However, concerns have arisen regarding its efficacy due to its transient nature, distracting attributes, and the cognitive overload it imposes on working memory [6,8,14].

Animations have been indirectly studied in electronic book formats for instructing young children [18,19,20,21]. Many studies associate animations with children’s vocabulary and narration, but they primarily rely on multimedia approaches [9]. Consequently, they are unable to offer clear insights specifically on animation due to the interference of other variables. Beyond the positive or negative results reported in these studies, they also reflect children’s familiarity with technology from an early age [22].

### 1.2. Animation in Speech and Language Pathology Research

In the field of SLP, static pictures are commonly used as visual stimuli for language-related tasks, while the potential utility of animation has received limited research attention. To the best of our knowledge, only a few studies have explored the effect of animation versus static pictures in young TD children, and this is primarily within the field of augmentative and alternative communication (AAC).

AAC employs various symbols to represent client needs and communication concepts and facilitates children with developmental disabilities “to understand, produce, and learn language” [23] (p. 1). Iconicity, the visual relationship of symbols to their referents (visual resemblance), is categorized by the ease of guessing their meanings: transparent (easily guessed), translucent (obvious with context), and opaque (not readily perceived) [24]. The aim of AAC is to enhance users’ communicative competence, encompassing linguistic, operational, social, and strategic abilities, as well as psychosocial and environmental factors [25,26].

Jagaroo and Wilkinson [27] discussed the role and function of motion perception from the perspective of cognitive neuroscience, arguing for the use of animated symbols in AAC and presenting a theoretical framework to guide speech–language pathologists’ clinical decisions. Frick et al. [2] reviewed previous research on animation in AAC and suggested potential applications of animation to enhance linguistic and operational competence for individuals with complex communication needs. In the field of the brain–computer interface in AAC (BCI-AAC), Pitt et al. [28] investigated the use of functional animated symbols in children aged 9 to 13 years old, reporting preliminary positive results. Finally, Schlosser et al. [29] conducted a scoping review that describes the roles of animation in AAC for TD individuals and those with developmental disabilities (children and adults).

In the field of AAC, studies comparing animations to static pictures as symbols in TD children were conducted by Mineo et al. [30], Schlosser et al. [31,32], Gunduz [33], and Brock et al. [23]. Additionally, Harmon et al. [34] investigated animated symbols alongside environmental sounds.

Also, Frizelle et al. [35] explored the effect of animation compared to static pictures on TD children’s language development using animations to assess complex syntax. Finally, Diehm et al. [36] investigated children’s narrative retelling skills in response to an animated video story versus a static pictures book, employing a multimedia features approach.

Considering all of the above, there is a significant gap in the literature regarding the effect of animation on young children’s typical language development.

### 1.3. Children’s Language Development

Language is considered a behavior that involves comprehension and use. It is typically described as spoken and written (or other communication symbol systems). These two forms include receptive (listening and reading) and expressive (speaking and writing) components. Language also comprises of five domains: phonology, morphology, syntax, semantics, and pragmatics [37].

Speech language pathologists (SLPs) do not apply instructional approaches when working on language development with young children for several reasons: (a) Language development cannot be taught as a set of rules [38]. (b) Assessment and/or intervention is individualized. (c) The primary goal of intervention is to enhance language skills and communication [39]. (d) Formal literacy skills are not fully developed or may still be emerging [40]. (e) Various language aspects or progress are evaluated using specific standardized tests [41]. Consequently, SLPs cannot adapt and apply instructional practices designed for adult learners to children. Finally, it should be noted that both language and cognitive abilities are under development throughout this stage of life [42,43], emphasizing the need for specific research tailored to this population.

Given these considerations, it is necessary to investigate whether animation serves as an effective tool for fostering language development in children.

### 1.4. The Present Study

Twenty years ago, Tversky et al. described animation as an “attractive graphic device” [8] (p. 250). However, the authors raised doubts about the efficacy of animation in instruction and its ability to convey concepts described with many words or to externalize internal knowledge. This prompts us to question whether animation presentations have positive or negative effects on the language development of young children, and if so, how do these effects manifest.

The primary objective of this systematic review is to gather evidence of spoken language responses (receptive—listening or expressive—speaking) in TD children exposed to a brief animated stimulus. A secondary aim is to document any other reported non-verbal behaviors (e.g., motivation, concentration, and distraction).

The following research questions were asked:

1. Can animation better support children’s receptive language abilities compared to a static picture?
2. Does animation enhance children’s expressive language more than a static picture?

This systematic review is necessary because there are a lack of data on how animation impacts children’s language development in SLP literature. The findings are expected to contribute to future research questions and suggestions for clinical practice.

## 2. Methods

The present systematic review is based on the preferred reporting items for systematic reviews and meta-analyses protocol (PRISMA) and the relevant flow diagram [44,45]. This review was not pre-registered.

### 2.1. The Present Study

The selection criteria for the included studies were established following the PICO model (population, intervention, comparison, and outcome) as described in Table 1 [46].

The focus centered on studies involving TD children within the specific age range to establish a basis for this population. Papers should include brief animations lasting less than one minute. The studies should either compare animations to static pictures or compare other animation characteristics. The sentence level was defined as the maximum linguistic response.

In terms of outcomes, the goal was to report data on language responses examined in each study, as well as language responses reported as secondary to the main study objectives. Furthermore, it was meaningful to record any other reported behaviors, whether positive or negative, potentially triggered by the presence of animation.

A brief animation was defined as the minimum duration from which evidence would be extracted. The main research purpose of the included papers should be animation involving some aspect of TD children’s language (receptive or expressive). Studies from the instructional field, and those related to e-books, applications, and television viewing were excluded.

### 2.2. Information Sources

A literature search from October to December 2022 was conducted by the first author, considering selection criteria covering the past 20 years (2002–2022). The databases Scopus, Web of Science, PubMed, ScienceDirect, and the Educational Resources Information Center (ERIC) were searched.

Due to the limited availability of relevant studies, further research was conducted by examining reference lists and the journal *“Computer Animation and Virtual Worlds”*, and consulting Google Scholar up to February 2023. In both search avenues, articles, conference papers, and theses that employed quantitative research methods were included.

### 2.3. Search Strategy and Eligible Studies

Different combinations of the following search terms were used: “animation”, “language”, “preschool children”, “language development”, “vocabulary”, “verbs”, “learning”, “animated books”, and “animated pictures”.

The search process involved standard steps: identification, screening (title, abstract, and full text), and inclusion of the final studies. Initially, 5165 studies were identified across all databases. After applying filters based on time and scientific areas, and removing duplicates and ineligible records, 51 studies remained. During the title screening, 8 studies were excluded (other reviews and meta-analyses), leaving 43. Subsequently, 22 more studies were excluded after reviewing the abstracts (qualitative studies or ineligibility based on inclusion criteria), leaving 21. In the full-text reading stage, 17 studies were excluded because of their indirect relevance to the research questions. Ultimately, only 4 studies were included.

Additional searches, including reference lists and Google Scholar, uncovered 31 studies. Four were excluded based on title content (reviews and meta-analyses), and twelve more were excluded due to differing research approaches. After reviewing the remaining 15 studies, 12 were excluded for indirect animation investigation or different approaches. The second search added 3 studies to the systematic review, resulting in a total of 7 included studies.

**Figure 1:**
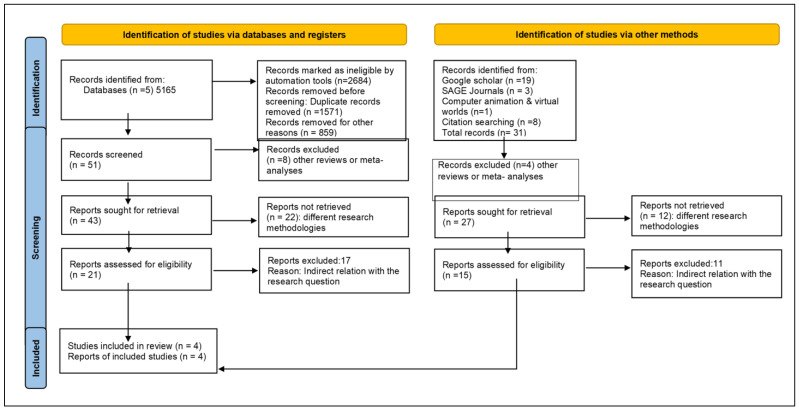
PRISMA flow diagram.

The complete process is illustrated in Figure 1, using the PRISMA 2020 flow diagram [44,45] for new systematic reviews, encompassing searches across databases, registers, and other sources.

The studies meeting the inclusion criteria were as follows: Mineo et al. [30], Schlosser et al. [31], Schlosser et al. [32], Harmon et al. [34], Gunduz [33], Frizelle et al. [35], and Brock et al. [23].

## 3. Results

### 3.1. Aims of the Eligible Studies

The key attributes of the included studies, encompassing research design, research purpose, participants, animated items, comparisons, and animation-related outcomes (positive, no difference, and negative) are presented in Table 2.

The first study investigating the effect of animation in the field of AAC was conducted by Mineo et al. in 2008 [30]. They employed a within-group design to examine the identification of 24 verbs across four conditions (video, animation, static line drawings with disequilibrium cues, and static line drawings with movement cues) in a sample of 93 TD children aged 3, 4, and 5 years. The results indicated that children performed more effectively in dynamic conditions (i.e., video and animation) as opposed to static conditions. Additionally, a developmental effect was observed, with symbol recognition improving as children grew older.

Schlosser et al. [31] used a mixed-group design to examine transparency, name agreement, and identification of 24 animated verbs and 8 animated prepositions compared to their corresponding static symbols with 53 TD children aged 3, 4, and 5 years. The results showed a positive effect on transparency and name agreement in verbs, with no significant difference found for prepositions. Additionally, no significant difference was observed in the identification task of verbs and prepositions. A similar developmental effect to the study by Mineo et al. [30] was evident in all measures.

Schlosser et al. [32] employed a randomized 2 × 2 × 2 × 3 factorial mixed design to investigate the naming and identification of 24 animated verbs and 8 animated prepositions in comparison to their static versions. Additionally, this study compared two symbol sets: the Autism Language Program [ALP] developed by Boston Children’s Hospital (https://www.childrenshospital.org/programs/autism-language-program (accessed on 27 August 2023)) [47] and the Picture Communication System [PCS] [48]. This study featured the largest sample, consisting of 220 TD children aged 3, 4, and 5 years. The results showed positive outcomes for naming verbs and prepositions, with no significant difference in identification. Furthermore, children exhibited better performance with ALP symbols over PCS symbols, and the developmental effect was reaffirmed.

In 2014, Harmon and colleagues [34] examined whether the addition of environmental sounds (e.g., breaking and door opening) improves the comprehension of animated symbols. They employed a between-group design, comparing 18 animated verbs with and without environmental sounds in a naming task. They recruited a sample of 46 TD children aged 3; 1–3; 11 years, who were randomly assigned to two groups. The results revealed a significant increase in naming accuracy when symbols were presented with environmental sounds.

Gunduz [33] replicated Schlosser et al.’s [32] study using a sample of 97 TD Turkish-speaking children aged 3, 4, and 5 years in Turkey. A 2 × 2 × 2 × 3 randomized factorial design was employed to investigate the naming and identification of 24 animated verbs and 8 animated prepositions using the same two sets of symbols (ALP and PCS). The key findings were replicated as follows: (a) naming accuracy was higher for animated verbs than for prepositions, (b) naming accuracy was superior for ALP animated symbols, (c) the developmental effect of animation was once again observed, and (d) no significant difference was found in the identification task.

In 2019, Frizelle et al. [35] compared two assessment methods for the understanding of complex syntax. They examined a sentence verification animation task and a multiple-choice picture selection task within a sample of 103 TD children aged 3; 6 to 4; 11 years. The study included five types of relative clauses, and the findings indicated that children exhibited superior performance in the sentence verification animation task.

Brock et al. [23] utilized a counterbalanced 2 × 2 × 2 mixed design to investigate the effect of animated symbols on forming sentences. They conducted identification and labeling tasks for 21 sentences with a sample of 24 TD children (aged 7; 0–8; 11 years). Additionally, they introduced a psycholinguistic analysis considering word frequency, imageability, and concreteness. The findings revealed a positive effect of animation on identification and labeling accuracy, primarily for verbs and prepositions, along with a reduction in the psycholinguistic effects of word frequency and imageability.

Most AAC studies have explored the iconicity of single animated symbols (e.g., [30,31,32,33]. These experiments compared animated symbols to static symbols, while only Harmon et al. [34] compared animated symbols with and without environmental sounds. Additionally, Brock et al. [23] and Frizelle et al. [35] focused on the sentence level (see Table 2).

In summary, all of the AAC studies mentioned follow a chronological progression in animation research employing consistent research methods, enabling meaningful comparisons. It is important to note that these studies were carried out at an experimental level, not a clinical one, and all report positive results for animation effect.

### 3.2. Quality Appraisal of Studies

The eligible studies are reported as experimental by the authors and included typically developing (TD) children as participants. The researchers aimed to investigate new variables [23] to establish a research base for children’s developmental capacities in response to animation before applying it in clinical populations [30,32,33,34,35]. Consequently, the group research designs used did not involve clinical and control groups. Randomization was employed in presentation order or to assign participants to different groups or conditions, and children were informed by the researchers that they were playing a computer-based game. Given these circumstances, two research quality appraisal methods were employed to ensure completeness and fairness.

In the appraisal process, the first and fourth authors independently assessed each study, resolving any discrepancies in discussion with the second author.

Initially, the PEDro-P scale for randomized (RCTs) and non-randomized control trials (NRCTs) was applied (https://speechbite.com/group-comparison-studies (accessed on 18 July 2023)). The PEDro-P scale derives from the original PEDro scale [49], with its reliability investigated by [50]. Both scales consist of 11 criteria evaluating various bias risks, resulting in a final score ranging from 0 to 10. PEDro-P differs in the definition and application of criterion 4. Although the total score of the original PEDro scale lacks validation, it is widely accepted as 0–3 for ‘poor’, 4–5 for ‘fair’, 6–8 for ‘good’, and 9–10 for ‘excellent’ evidence quality (https://pedro.org.au (accessed on 20 July 2023)). The authors sought guidance from the speechBITE training program. The PEDro-P ratings for the studies are presented in Table 3.

Also, the quality assessment tool for quantitative studies (QAT) was employed (https://www.ephpp.ca/quality-assessment-tool-for-quantitative-studies (accessed on 18 July 2023)). QAT is a result of the Effective Public Health Practice Project (EPHPP) initiated by the Ontario Ministry of Health [51]. This tool evaluates the following factors: (a) selection bias, (b) study design, (c) confounders, (d) blinding, (e) data collection methods, (f) withdrawals and dropouts, (g) intervention integrity, and (h) analyses. The quality of evidence is categorized as 1 for “strong”, 2 for “moderate”, and 3 for “weak”. The final rating is “strong” if no “weak” ratings are present, “moderate” if one “weak” rating is identified, and “weak” if two or more “weak” ratings are found. The authors referenced the QA dictionary, and the QAT ratings are presented in Table 4.

As shown in Table 3 and Table 4, all studies received a score of 7 on the PEDro-P scale and were rated as “strong” in quality on the QAT, except for the study by Brock et al. [23], which was rated as “moderate”. Consequently, both tools suggest a good to moderate-to-strong level of evidence. The research quality of the included studies is further discussed below.

Regarding the eligibility criteria and selection bias, the authors targeted specific samples with specific characteristics from the typical population for research purposes. Only Frizelle et al. [35] applied an opt-out protocol to minimize volunteer bias. Sample sizes ranged from 46 to 220 subjects, except for the study by Brock et al. [23], which included 24 children (see Table 2). Additionally, some studies performed power calculations [31,32,35]. All studies employed both standardized and informal tests to assess children before their participation in research projects. Withdrawals and dropouts were documented in [34,35].

Randomization was applied in [32,33,34]. Mineo et al. [30], Schlosser et al. [31], Frizelle et al. [35], and Brock et al. [23] randomized the presentation order of items and/or the order of conditions. None of the studies reported concealed allocation, likely because they did not involve clinical populations or therapy methods.

Furthermore, all of the potential nuisance variables were controlled, and the research limitations were reported by the authors. The study by Brock et al. [23] faced a unique limitation due to the COVID-19 pandemic, necessitating the use of the Zoom video conferencing tool. This is reflected in the “moderate” rating of QAT.

Most studies conducted complete statistical analyses. However, some studies provided additional insights through error analysis (e.g., [30,34]), cross-linguistic analysis [33], or psycholinguistic analysis [23], in line with their research objectives. Frizelle et al. [35], in their comparison of two syntax comprehension tasks for assessment purposes, employed a standardized test to validate their findings.

In all studies, blinding of researchers was not applied, likely due to the introduction of research tasks on a computer or tablet, which enhanced the validity and fidelity of administration and scoring. As a result, criterion 7 on the PEDro-P scale was marked as “Not Applicable” (NA). Also, prior to the main research phase, children had the chance to acquaint themselves with the software and computer or tablet demands.

In the study of Frizelle et al. [35], it was reported that the research was explained to the children without providing additional details by the authors. In the AAC studies, subjects were informed that they would participate in a guessing game. Since both cases involved young children who were not clinical populations, these methods were deemed blinding.

In conclusion, the eligible studies offer a sufficient level of evidence of the positive effect of animation on their respective research objectives.

### 3.3. Animations and Research Tasks

In consideration of the research questions, the characteristics of the animations and the relative tasks used in the included studies were recorded: (a) duration and number of animated items presented in each task, (b) realism level as classified by [14] (level 1—L1: schematic, level 2—L2: rather simple, level 3—L3: rather realistic, and level 4—L4: photo-realistic = video), (c) participants’ prior knowledge of the represented animated content (lexical items), (d) involvement of verbal linguistic components (receptive/expressive language) and/or domain (syntax, semantics, etc.), (e) target responses and participants’ responses (according to authors’ reports), coded as follows: no response (for receptive language), one or two words, or a phrase/sentence (for expressive language), (f) the language used in the research project, including potential reports for bilingual participants, and (g) reports on other observed behaviors of children (see Table 1). All of these characteristics are summarized in Table 5.

The durations of animations in the included studies varied from 6 to 29 s. Brock et al.’s [23] study lacks this specific information. Nevertheless, given their partial use of the same symbols as Schlosser et al. [31,32], it can be inferred that the duration falls within a similar range.

Regarding the number of animated items in each task, only the studies by Harmon et al. [34] and Frizelle et al. [35] presented a single animated item. Mineo et al. [30], Schlosser et al. [31,32], and Gunduz [33] introduced four and six animations concurrently (identification task), while Brock et al. [23] gradually introduced five animations within a sentence sequence.

All of the animations were created with colorful graphics designed for children in a two-dimensional (2D) format and their realism level varied from L1 to L2 [14]. Only in the study of Mineo et al. [30] did the authors create an experimental set of animated black and white line drawings (stick figures).

Most AAC studies utilized the same sets of animated symbols (ALP and PCS symbols) for different research objectives [31,32,33,34] that were not originally created for these studies. Schlosser et al. [32] and the replication study of Gunduz [33] compared these two sets of animated symbols (PCS and ALP) across two different languages. In contrast, Frizelle et al.’s [35] animations were designed specifically for their study. Lastly, Brock et al. [23] combined animated symbols from these two sets to construct sentences and introduced non-animated photographs in an identification task.

All studies in the field of AAC ensured that children possessed prior receptive or expressive knowledge of the lexical items (content of representation) being investigated, as they were exploring the level of iconicity of the symbols. On the other hand, Frizelle et al. [35] used animations as an assessment method to determine the presence or absence of the knowledge under examination (relative clauses).

Regarding language features, the included studies involved verbal forms (listening and speaking). All studies involved animated tasks that required receptive language demonstration, with five of them necessitating expressive language from children (see Table 5). Harmon et al. [34] introduced only animated expressive language tasks, adding environmental sounds in the control condition. Five studies [30,31,32,33,34] focused on the word level (animated verbs and/or prepositions), while two studies [23,35] addressed the sentence level (syntax). In these instances, one animation represented a complex sentence, or five animated symbols represented the sentence components, respectively.

The authors introduced animated symbols aiming for specific responses. In identification tasks (receptive language), no answer was required. The children simply had to select one animation from the others. In the case of Frizelle et al. [35], children had to verify if the animation was represented by the sentence that they heard. In naming or labeling tasks (expressive language), children had to produce one word for verbs, one or more words for prepositions (e.g., next to), or a sentence with five or more words (e.g., [23]) in response to a prompt question.

On the other hand, children’s expressive responses for verbs and prepositions varied from one word to a phrase or sentence containing the target word. Additionally, the authors reported that children sometimes used alternative words, such as synonyms and free morphemes [31,32,33,34]. Finally, in Brock et al.’s study [23], children demonstrated the ability to produce complete grammatical sentences or symbol-by-symbol labeling.

The English language predominated in the eligible studies, with only Gunduz [33] offering information on animated symbols in the Turkish language. Furthermore, Frizelle et al. [35] stands out as the sole study that explicitly mentions the exclusion of bilingual children. All other studies indicate that English is the primary language spoken at home.

Limited information was reported in the studies about other observed behaviors in children during the presentation of animations. Mineo et al. [30] (p. 169) noted that “… some children seemed somewhat agitated by the need to attend to multiple moving stimuli at the same time” and explained how children reacted and performed during the specific task. Harmon et al. [34] argue that even with the addition of environmental sounds, children seemed to rely more on the visual animated stimuli and reported that all participants visually attended to the screen. Finally, Frizelle et al. [35] reported that participants rarely requested a repetition of the animation for which they had to make a judgment.

In conclusion, apart from the environmental sounds in Harmon et al.’s [34] study, none of the animations featured simultaneous linguistic sounds (e.g., words). Essentially, most animations presented only visual stimuli, while Harmon et al.’s [34] study specifically focused on environmental, not linguistic, stimuli. The only instance of animation and a spoken sentence occurring concurrently was found in Frizelle et al.’s [35] study. Clearly, the choices made in each study were contingent upon their respective objectives and methodologies.

### 3.4. Focus on Aspects of Language

While studies in the field of AAC primarily focus on symbol research, the authors have addressed some language-related aspects in connection with their methodologies and related tasks. Thus, according to Mineo et al. [30], a language comprehension task (identification) was employed to examine receptive language while comparing four forms of picture-based language representations. Similar tasks were also employed by Schlosser et al. [31,32] and Gunduz [33]. Furthermore, Schlosser et al. [31,32] argue that the identification task requires children to associate language with graphic symbols, and this adds a novel dimension to the study of animation.

On the other hand, tasks such as naming demanded expressive language from the participants. Schlosser et al. [32] discussed how this task triggered numerous alternative responses among the participants. Nevertheless, Harmon et al. [34] argued that the type of prompt used in these tasks might have influenced the children’s responses. Specifically, the instruction “What is this?” could lead to responses containing nouns or sentences consisting of both nouns and verbs, rather than the desired verb. Eventually, Harmon et al. [34] suggested that the training tasks aided the participants in grasping the experimental task objectives. However, it remains unclear whether this phenomenon (i.e., the production of sentences) was exclusive to animated stimuli or also occurred with the static items (e.g., [31,32,33]). This question is essentially addressed in the Harmon et al. [34] study, which exclusively involved animated stimuli and arrived at similar observations.

Harmon et al.’s [34] analysis of semantic errors of children sheds light on a distinct aspect of language. The researchers observed semantic type superordinate errors (among others) across conditions (i.e., animations without sound vs. animations with environmental sounds) where the trend was to name the animated symbol with a more general verb instead of the target verb. The authors purported that this finding is consistent with the children’s early vocabulary development.

Gunduz [33] replicated the study of Schlosser et al. [32] by using the same animated symbols due to the unavailability of related symbols for the Turkish language. The author conducted a cross-linguistic analysis of Turkish by examining specific linguistic characteristics (e.g., word class, syntax, etc.) and suggested that the acquisition of verbs and prepositions may be language specific. The results (Table 2) generally aligned with those of Schlosser et al. [32], indicating that the animated symbols, without any adaptation, allowed for the representation of the same meanings in a different language, at least to some extent.

The study of Brock et al. [23] focused on syntax, utilizing both identification and labeling tasks involving animated symbols. Experimental sentences followed the subject–verb–object–prepositional phrase structure and were constructed by five animated symbols. As depicted in Table 5, children demonstrated the ability to follow this structure, generating grammatical sentences or symbol-by-symbol labeling, all without prior training. Furthermore, the authors introduced a novel dimension to AAC, conducting a psycholinguistic analysis that considered word frequency, imageability, and concreteness. They argued that animation reduced the effects of these factors.

Frizelle et al. [35] explored language assessment by testing children’s comprehension of complex sentences. They discovered that the sentence verification animation task enhanced children’s sentence comprehension abilities, offering a novel assessment approach. Additionally, they observed that each method revealed a different hierarchy of constructions and the impact of assessment method on the participants’ scores was greater for some constructions than others. Furthermore, the authors discussed the influence of pragmatically appropriate context on the children’s scores, as well as cognitive factors like attention and memory, which could affect scores in multiple-choice assessments using static pictures.

Finally, the evidence on bilingualism is severely limited. While Frizelle et al. [35] excluded bilingual children, the remaining included studies did not yield any clear indication of whether bilingual children were participants in the research.

## 4. Discussion

In this systematic review, we conducted a search for evidence regarding the effect of brief animations on the language development of TD children aged 3 to 9 years. This research topic was chosen due to the limited information available regarding the effectiveness of animations compared to static pictures in children’s language development. After a search and evaluation process, we identified seven relevant studies. One of these studies [35] focused on the use of animation to assess the understanding of complex syntactic structures. The remaining six studies [23,30,31,32,33,34] investigated the iconicity of animated symbols in the context of AAC. The studies included brief 2D animations (maximum duration of 29 s) with realism levels 1 and/or 2 [14] and involved a total of 636 TD children. The data collected from these studies are used to address our research questions.

For the first research question, “Can animation better support children’s receptive language abilities than a static picture?”, the primary source of evidence was the AAC studies. Although the core research objectives differed, five of the included studies [23,30,31,32,33] involved an identification task related to aspects of receptive language (see Table 5). That is, children were required to demonstrate their existing knowledge of the lexical item. Additionally, Frizelle et al. [35] directly addressed receptive language for sentences with complex syntax. However, the results vary, with Mineo et al. [30], Brock et al. [23], and Frizelle et al. [35] presenting positive findings, while Schlosser et al. [31,32] and Gunduz [33] found no significant difference between animated and static symbols for these tasks. Consequently, the evidence collected does not provide a conclusive answer to the first research question.

In addressing the second research question—“Does animation enhance children’s expressive language more than a static picture?”—the evidence rested upon the five studies from the field of AAC [23,31,32,33,34]. For diverse research objectives, these authors employed naming or labeling tasks where children were required to verbally express themselves following a verbal prompt while attempting to guess the meaning of the animated symbol. As detailed in Section 3.4, the participants’ responses regarding the length of utterance (number of words), alternative words (e.g., synonyms), semantic errors (vocabulary development), cross-linguistic replication (English to Turkish language), and the ability to produce complete grammatical sentences or perform symbol-by-symbol labeling without training [23] yielded valuable insights into expressive language abilities. This evidence suggests that animation may (a) enhance children’s expressive language skills, (b) indicate some aspects of language development, (c) exhibit cross-linguistic effects on word meaning, and (d) contribute to sentence production. These pieces of evidence provide a promising response to the second research question concerning the effect of animation on the expressive language of children.

### 4.1. Converging Results

Although Mineo et al. [30] and Frizelle et al. [35] conducted studies in different fields of SLP practice, both concur on the influence of assessment type and manner on the children’s performance. These studies employed animations for distinct research objectives, (AAC symbol identification and language assessment), with a shared focus on uncovering children’s receptive linguistic knowledge, addressing word knowledge and syntactical complexity, respectively. Mineo et al. [30] aimed to determine which form of visual stimulus (among four options) best facilitated children in demonstrating their lexical knowledge, while Frizelle et al. [35] explored whether two distinct assessment types (sentence verification animation task vs. multiple-choice picture selection task) yielded similar results when assessing comprehension of complex syntax. Both research teams designed their animations to suit their research objectives. In the study of Mineo et al. [30], participants already possessed lexical knowledge, while in Frizelle et al. [35], participants were undergoing assessment for the target knowledge. Furthermore, both studies compared their methodologies to those of standardized language test constructions (e.g., Peabody Picture Vocabulary Test—PPVT; Clinical Evaluation of Language Fundamentals—CELF4). Ultimately, despite their different research scopes, both studies reached a comparable conclusion regarding the positive impact of animation. This convergence of views and consistent positive results allow for some optimism for the potential future role of animation in SLP research and clinical practice.

### 4.2. Research and Clinical Implications

The current systematic review presents evidence of the effect of brief animations on the language development of TD children compared to static pictures. The only available data have been drawn from experimental studies within the field of SLP, rather than clinical investigations. Research concerning the application of animations in clinical populations fell outside the scope of this systematic review.

In this study, the minimum characteristics of brief animations concerning duration, design, and manner of presentation are outlined. These can serve as a valuable foundation for developing brief animations for clinical practice, particularly for children with language disorders. It is essential to highlight that apart from the positive outcomes revealing the effectiveness of animations, no adverse effects on the behavior of TD children were documented, which also is important as a basis for clinical studies.

### 4.3. Limitations and Future Research

The primary limitation of this systematic review is related to the scarcity of children’s language-related studies concerning brief animations compared to static pictures. The search process revealed only one study [35] that directly investigated the correlation between animation and language development of TD children. The AAC field provided important, albeit indirect, evidence concerning some aspects of receptive and expressive language knowledge through identification tasks and naming using animation. Overall, within the field of SLP, the AAC domain remains the most informed area of animation research over the past 15 years.

Speech–language pathologists can explore the various research pathways regarding the effects of animation on children’s language development (e.g., semantics, syntax, etc.), particularly for those facing language difficulties. Animation may facilitate the receptive or expressive language of children, but more research is needed (e.g., on the design of animation). Examining data from diverse language, cultural, and bilingual populations would be interesting. Nevertheless, the priority of this paper was to determine from the research evidence the most effective brief animation protocols for children acquiring their first language. This systematic review serves as a foundational step for future research.

The relationship between animation and cognitive load is a crucial topic that links duration, presentation speed, and the number of simultaneously moving stimuli to cognitive processing requirements and working memory limitations [6,7,8]. This study focused on brief animations, framing the minimum duration (under 30 s), speed, and realism level [8,14]. The findings revealed no adverse effects for a single animated item with these characteristics. However, the introduction of four, five, or six animations simultaneously on a screen may warrant further investigation. In the study of Mineo et al. [30], the viewing time for simultaneously introduced animations was the shortest compared to other studies included in Table 2. The authors argued that this format is like other standardized language tests (e.g., PPVT). Conversely, Frizelle et al. [35] discussed how the same style of standardized language tests with static pictures may impose on cognitive load.

Furthermore, Schlosser et al. [32] and Gunduz [33] actually compared two animated symbol sets with different realism levels. Also, Schlosser et al. [32] argued that the identification task (forcing a choice between four animations) was easier compared to the naming task (naming a single animated symbol). Finally, Brock et al. [23] discussed the practical implications of simultaneously looped animations and suggested modifications related to user control. Therefore, future research on methods for introducing animations should consider the cognitive abilities under development and the target populations for assessment or intervention.

## 5. Conclusions

The present systematic review investigated the effect of brief animations on language development of typically developing (TD) children compared to static pictures. The characteristics of the animations, the tasks used, and the children’s responses were recorded. The evidence collected on verbal language responses suggested that animations may positively affect expressive language. However, the evidence regarding receptive language is less conclusive. While our findings permit some optimism, further research specifically focused on the effect of animations on children’s language development is necessary.

## Figures and Tables

**Table 1:** Inclusion and exclusion criteria.

Selection Criterion	Description
**P**opulation/problem	TD children 3 to 9 years old
**I**ntervention	Presentation of brief animations (under one minute duration) individually provided by SLPs or psychologists regardless of the frequency of administration.
**C**omparisons	Animations versus static pictures or comparisons of different animation features.
**O**utcomes	Language response: (a) targeted within the research; (b) reported but not targeted. Other behaviors: (a) wanted (e.g., attention, concentration, participation, and motivation) or (b) undesired (e.g., distraction, withdrawal, frustration, and overuse).
Settings	Research clinic, schools, nurseries, and in the home.
Limits	Animation is the main research purpose which involves some language aspect. Studies published in English language without local restrictions.
Exclusion criteria	Multimedia applications and e-books. Research that examines animations in adults and non-typically developing children. Animated stories aimed at narration. Studies using animation as feedback and/or reward. Papers regarding instruction and television animation viewing.

**Table 2:** Studies’ characteristics.

Study	Research Design	Research Purpose/Animation Function	Participants	Animated Items	Comparisons	Outcomes Regarding Animation
Mineo et al. [30]	Within-group design	Iconicity of symbols—identification	93 TD children (3-, 4-, and 5-years old)	24 verbs	Four conditions: video—animation—static/dis—static/move * symbols	Positive for identification of verbs.
Schlosser et al. [31]	Mixed-group design	Iconicity of symbols—transparency, name agreement, and identification	53 TD children (3-, 4-, and 5-years old)	24 verbs and 8 prepositions	Animated vs. static symbols	Positive for transparency and name agreement in verbs, no difference in prepositions, and no difference for identification in verbs and prepositions.
Schlosser et al. [32]	Randomized 2 × 2 × 2 × 3 factorial mixed design	Iconicity of symbols—naming and identification	220 TD children (3-, 4-, and 5-years old)	24 verbs and 8 prepositions	Animated vs. static/two sets of symbols	Positive for naming in verbs and prepositions/no difference for identification.
Harmon et al. [34]	Between-group design	Iconicity of symbols—naming	46 TD children (3; 1–3; 11 years old)	18 verbs	Animated symbols with environmental sounds vs. without sounds	Positive for naming mostly in environmental sounds condition.
Gunduz [33]	2 × 2 × 2 × 3 randomized factorial design	Iconicity of symbols—naming and identification	97 TD children (3-, 4-, and 5-years old)	24 verbs and 8 prepositions	Animated vs. static/two sets of symbols	Positive for naming mainly in verbs/no difference for identification.
Frizelle et al. [35]	Within-group design	Assessment of syntax understanding—language comprehension ability	103 TD children (3;6–4;11 years old)	50 sentences (five types of relative clauses)	Sentence verification animation task vs multiple-choice picture selection task	Positive for syntax understanding.
Brock et al. [23]	Counterbalanced 2 × 2 × 2 mixed design	Iconicity of symbols—identification and labeling	24 children (7;0–8;11 years old)	21 sentences	Animated vs. static symbols and psycholinguistic effects	Positive for identification and labeling accuracy mainly in verbs and prepositions/animation reduced the psycholinguistic effects (word frequency and imageability),

* Static/dis: static line drawings with disequilibrium cues; static/move: line drawings with movement cues.

**Table 3:** The PEDro-P ratings out of 10.

Study	1	2	3	4	5	6	7	8	9	10	11	Total Score
Mineo et al. [30]	1	1	0	1	1	0	NA *	1	1	1	1	7/10
Schlosser et al. [31]	1	1	0	1	1	0	NA	1	1	1	1	7/10
Schlosser et al. [32]	1	1	0	1	1	0	NA	1	1	1	1	7/10
Harmon et al. [34]	1	1	0	1	1	0	NA	1	1	1	1	7/10
Gunduz [33]	1	1	0	1	1	0	NA	1	1	1	1	7/10
Frizelle et al. [35]	1	1	0	1	1	0	NA	1	1	1	1	7/10
Brock et al. [23]	1	1	0	1	1	0	NA	1	1	1	1	7/10

* NA: not applicable.

**Table 4:** The QAT Ratings.

Study	Selection Bias	Study Design	Confounders	Blinding	Data Collection Method	Withdrawals and Dropouts	Final Rating
Mineo et al. [30]	Strong	Moderate	Strong	Moderate	Strong	Strong	Strong
Schlosser et al. [31]	Strong	Moderate	Strong	Moderate	Strong	Strong	Strong
Schlosser et al. [32]	Strong	Strong	Strong	Moderate	Strong	Strong	Strong
Harmon et al. [34]	Strong	Strong	Strong	Moderate	Strong	Strong	Strong
Gunduz [33]	Strong	Strong	Strong	Moderate	Strong	Strong	Strong
Frizelle et al. [35]	Strong	Moderate	Strong	Moderate	Strong	Strong	Strong
Brock et al. [23]	Moderate	Moderate	Strong	Moderate	Weak	Strong	Moderate

**Table 5:** Characteristics of animations and research tasks.

	Mineo et al. [30]	Schlosser et al. [31]	Schlosser et al. [32]	Harmon et al. [34]	Gunduz [33]	Frizelle et al. [35]	Brock et al. [23]
**Duration**	10 s/30 frames per second	14 s transparency; 20 s identification	14 s naming; 20 s identification	13–29 s	14 s naming; 20 s identification	6 s average	No report
**Animations presented per task**	4	1 or 4	1 or 4	1	1 or 6	1	5
**Realism level**	L1 *	L2	L1 and L2	L2	L1 and L2	L2	L2
**Previous knowledge of the items**	YES	YES	YES	YES	YES	Under assessment	YES
**Verbal linguistic component(s) involved and/or domain**	Receptive language (verbs)	Receptive/expressive language (verbs and prepositions)	Receptive/expressive language (verbs and prepositions)	Expressive language (verbs)	Receptive/expressive language (verbs and prepositions)	Receptive language/syntax (relative clauses)	Receptive/expressive language (sentences) and psycholinguistic characteristics
**Target responses/** **number of words**	No word	One word for verbs and 1–2 words for prepositions	One word for verbs and 1–2 words for prepositions	One word	One word for verbs and 1–2 words for prepositions	No word	A sentence (five or more words)
**Responses of the participants**	No response was demanded	One word, an alternative world, a phrase, or sentence	One word, an alternative word, a phrase, or sentence	One word, an alternative word, a phrase, or sentence	One word, an alternative word, a phrase, or sentence	No response was demanded	Complete grammatical sentences or symbol-by-symbol labeling
**Language used**	English	English	English	English	Turkish	English	English
**Bilingualism reported**	NO	ΝO	NO	NO	NO	Bilinguals excluded	NO
**Other behaviors**	Viewing response to simultaneous animated stimuli	No report	No report	Visual attention to the screen	No report	Rarely requested animation repetition	No report

* L: level.

## Data Availability

No new data were created.
